# Cohort effects in dynamic models and their impact on vaccination programmes: an example from Hepatitis A

**DOI:** 10.1186/1471-2334-6-174

**Published:** 2006-12-05

**Authors:** Arni SR Srinivasa Rao, Maggie H Chen, Ba' Z Pham, Andrea C Tricco, Vladimir Gilca, Bernard Duval, Murray D Krahn, Chris T Bauch

**Affiliations:** 1 Department of Mathematics and Statistics, University of Guelph, Guelph, Canada; 2 Toronto General Hospital and University Health Network, Toronto, Canada; 3 Department of Health Policy, Management and Evaluation, University of Toronto and University Health Network, Toronto, Canada; 4 Institut national de santé publique Québec, Québec City, Canada, and Research Centre, Centre hospitalier universitaire de Québec, Québec City, Canada; 5 BioMedical Data Sciences, GlaxoSmithKline, Toronto, Canada

## Abstract

**Background:**

Infection rates for many infectious diseases have declined over the past century. This has created a cohort effect, whereby older individuals experienced a higher infection rate in their past than younger individuals do now. As a result, age-stratified seroprevalence profiles often differ from what would be expected from constant infection rates.

**Methods:**

Here, we account for the cohort effect by fitting an age-structured compartmental model with declining transmission rates to Hepatitis A seroprevalence data for Canadian-born individuals. We compare the predicted impact of universal vaccination with and without including the cohort effect in the dynamic model.

**Results:**

We find that Hepatitis A transmissibility has declined by a factor of 2.8 since the early twentieth century. When the cohort effect is not included in the model, incidence and mortality both with and without vaccination are significantly over-predicted. Incidence (respectively mortality) over a 20 year period of universal vaccination is 34% (respectively 90%) higher than if the cohort effect is included. The percentage reduction in incidence and mortality due to vaccination are also over-predicted when the cohort effect is not included. Similar effects are likely for many other infectious diseases where infection rates have declined significantly over past decades and where immunity is lifelong.

**Conclusion:**

Failure to account for cohort effects has implications for interpreting seroprevalence data and predicting the impact of vaccination programmes with dynamic models. Cohort effects should be included in dynamic modelling studies whenever applicable.

## Background

### Hepatitis A

Hepatitis A virus (HAV) is endemic in most countries [1-4], although infection rates continue to decline as hygiene and sanitation improve [5]. Infection is often subclinical in children but the majority of infected adults develop clinical symptoms, and often do not seek medical attention [5,6]. The severity of HAV infection increases with age and is particularly severe for those with underlying chronic liver disease [7-10]. The rate of mortality attributable to HAV increases by age from a 0.2% in symptomatic young adults to 3.9% in symptomatic adults over the age of 80 [11]. The clinical illness usually lasts 4 weeks and symptoms include nausea, loss of appetite, fatigue, fever, abdominal pain and jaundice [6].

HAV is transmitted by the fecal-oral route. Chronic infection does not occur [12], and immunity appears to be lifelong [7]. Traditionally, children have played a prominent role in transmission due to poor hygiene and the high probability of subclinical infection [13]. The reported incidence in Canada from 1980–1994 was twice as high in the 5–9 age groups as in other age groups [14], and once the reduced probablility of clinical infection in children is taken into account [6,15-19], true infection rates in children are seen to be significantly higher than in adolescents and adults. Approximately 25–50% of clinical cases have unidentified risk factors [20]. Many of these may have been contacts of children with subclinical infection.

**Figure 1:**
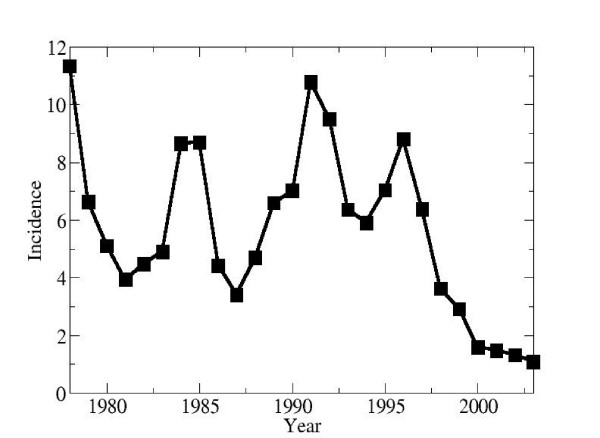
Hepatitis A incidence in Canada (number of cases per 100,000 per year), 1978–2003.

In Canada, HAV is not endemic and transmission is primarily person-to-person [5] with rare foodborne outbreaks [21]. Risk factors for contracting HAV are often unidentified [20,21], but known risk factors include membership in high risk groups (including men who have sex with men, injection drug users and the homeless), low socioeconomic conditions [22], membership in socially-contained religious communities [23-25], and travel to endemic areas [26,27]. The latter is a particularly significant risk factor in Canada. Secondary infections from index cases infected abroad [25,28,29] and intra-household transmission [30] are also significant. From 1980 to 1994 (the year of vaccine licensure in Canada), the average incidence of reported cases was 6.3 per 100,000 per year [14] (Figure 1).

Case reports reveal when clinical infections occur, but they do not account for subclinical infection and under-reporting [1,5]. By comparison, seroprevalence data can accurately determine what proportion of individuals have been infected by a given time, but cannot say when the infection occurred. Estimates of true infection rates usually rely upon seroprevalence surveys and/or case reporting data. Catalytic modelling uses integral equations to reconcile case reporting and seroprevalence data via statistical regression, yielding estimates of the true infection rates and how they have evolved over time. According to estimates from catalytic modelling, infections are under-reported by a factor of 10 in the United States [31,32].

### Cohort effects

To date, there is no representative national seroprevalence survey for HAV in Canada. However, an approximate age-stratified seroprevalence profile has recently been synthesized from various independent surveys by the method of systematic review [33]. The resulting profile is both reasonable and consistent with the profile observed in the NHANES (National Health and Nutrition Examination Survey) II and III surveys in the United States [31]. The observed seroprevalence in Canada and the United States is too low in the youngest age classes and too high in older age classes to be consistent with the assumption of constant infection rates over time. Instead, it is necessary to posit that infection rates were higher in past decades due to a lower level of sanitation and hygiene [34]. This is usually described as a cohort effect [5]. Cohort effects have also been observed in other populations [34,35].

**Figure 2:**
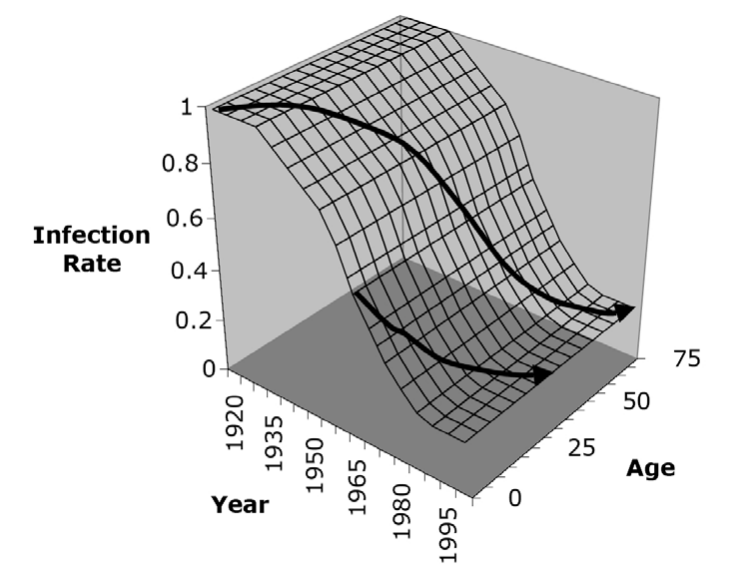
**Illustration of the cohort effect for an idealized situation of no age dependence in infection rates**. The top arrow represents time evolution in the infection rate on a cohort born in 1920 and the bottom arrow represents the same for a cohort born in 1970. The infection rate is the rate at which a susceptible person becomes infected, and here it has been expressed in an idealized unit of measurement.

The presence of cohort effects in seroprevalence data is widely recognized. However it is difficult to characterize cohort effects without a mathematical model since the two dimensions involved – age class and calendar year – are easily confounded. Figure 2 illustrates how two cohorts born at different times experience different infection rate histories, when infection rates decline over time. The expected seroprevalence for a given cohort can be thought of as an integration of the infection rate history over the cohort's life history path in Figure 2. When infection rates have been constant over time, age-stratified seroprevalence profiles have a convex shape, increasingly monotonically with age but with progressively smaller increases for increasing age. However, when a strong cohort effect is present due to rapidly declining infection rates, an S-shaped age-stratified seroprevalence profile should be observed.

### Goals of study

Few dynamic models have been developed for HAV, compared to other common infectious diseases [34,36-38]. Dynamic modelling studies and other studies which estimate infection rates for many diseases often do not take the cohort effect into account, resulting in inaccurate predictions. Here we develop and analyze a dynamic model for Hepatitis A in Canada. Our goal is to quantify how infection rates have declined over the past century, to illustrate how dynamic models which include the cohort effect can reconcile case reporting data and seroprevalence data, and to illustrate how the predicted impact of vaccination on incidence and mortality varies depending on whether the cohort effect is accounted for in the dynamic model.

## Methods

### Compartmental model

We develop an age-structured compartmental model to study HAV transmission and vaccination in Canada. The literature on age-structured compartmental models is well-established and has developed extensively since the mid-1980s [39]. Such models have long been applied to infectious diseases where natural immunity is lifelong and transmission is primarily person-to-person, as is the case for HAV in Canada. They have been validated against epidemiologic data [39-42] and have been used by health authorities in decision making processes for vacination policy [43]. Our SEIRV (Susceptible-Exposed-Infectious-Recovered-Vaccinated) compartmental model allocates all members of the population into mutually exclusive compartments according to age class and epidemiologic status: the number susceptible (*S*_*i*_), exposed (infected but not yet infectious, *E*_*i*_), infectious (*I*_*i*_), recovered (*R*_*i*_), and vaccinated (*V*_*i*_) in each age class *i*. The age classes are 0–4, 5–9, 10–19, 20–29, 30–39, 40–59 and 60+, chosen to reflect age categories in available sources of demographic and epidemiologic data.

**Figure 3:**
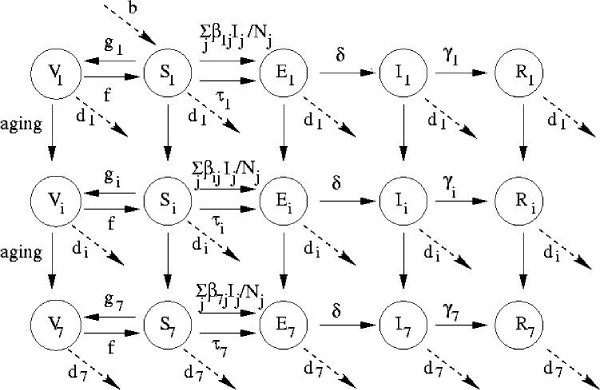
Diagram of the SEIRV model.

Flow rates between compartments are defined by model parameters. Exposed individuals enter the infectious compartment at rate *δ*, and infectious individuals enter the recovered compartment at rate *γ*_i_, thereafter retaining lifelong immunity. Individuals in age class *i *are vaccinated at per capita rate *g*_*i *_thereby entering the vaccinated compartment, and vaccinated individuals lose their immunity at per capita rate *f*, re-entering the susceptible compartment. Individuals are born susceptible at rate *b*, and when a given birth cohort enters the next highest age class, a proportion *d*_*i *_of them die. Maternal immunity is short-lived and affects relatively few individuals in a non-endemic country such as Canada, so we do not include it [5]. HAV in Canada is spread primarily person-to-person [5]. Hence we do not model foodborne or waterborne outbreaks. The transmission rates and the model for the cohort effect are described in the following subsections. The model is parameterized using data from the clinical literature [44-50], demographic [51] and travel data [26,52-55], case reports [14], and seroprevalence suveys [33]. Model equations appear in Additional File 1 and the parameterization is described in Additional File 2. A diagram of the model appears in Figure 3.

### Travel transmission rates

Estimates of the proportion of HAV cases attributable to travel in endemic countries range from 26% to 40%, depending upon the year and location [26,53-55]. A considerably higher proportion of cases are attributable to travel in younger age classes than in older age classes [55]. The predicted impact of vaccination, and the fit of the model to the data, can vary significantly depending on whether travel-related incidence is accounted for. Here we include travel-related incidence in our model.

If *λ*_*i *_denotes the total infection rate in age class *i *(the rate at which a susceptible individual in age class *i *becomes infected), and if *κ*_*i *_denotes the proportion of infections in age class *i *attributable to travel in endemic countries, then clearly *τ*_*i *_= *κ*_*i *_*λ*_*i *_where *τ*_*i *_is the travel transmission rate, ie, the rate at which a susceptible person in age class *i *becomes infected due to travel in an endemic country. Let τ¯
 MathType@MTEF@5@5@+=feaafiart1ev1aaatCvAUfKttLearuWrP9MDH5MBPbIqV92AaeXatLxBI9gBaebbnrfifHhDYfgasaacH8akY=wiFfYdH8Gipec8Eeeu0xXdbba9frFj0=OqFfea0dXdd9vqai=hGuQ8kuc9pgc9s8qqaq=dirpe0xb9q8qiLsFr0=vr0=vr0dc8meaabaqaciaacaGaaeqabaqabeGadaaakeaaiiGacuWFepaDgaqeaaaa@2E90@_*i *_be the average travel transmission rate for 1980–1994. We estimate τ¯
 MathType@MTEF@5@5@+=feaafiart1ev1aaatCvAUfKttLearuWrP9MDH5MBPbIqV92AaeXatLxBI9gBaebbnrfifHhDYfgasaacH8akY=wiFfYdH8Gipec8Eeeu0xXdbba9frFj0=OqFfea0dXdd9vqai=hGuQ8kuc9pgc9s8qqaq=dirpe0xb9q8qiLsFr0=vr0=vr0dc8meaabaqaciaacaGaaeqabaqabeGadaaakeaaiiGacuWFepaDgaqeaaaa@2E90@_*i *_from the average force of infection *λ*_*i *_from 1980–1994 as estimated by catalytic modelling, and from average *κ*_*i *_values for 1980–1994, estimated from surveys on HAV risk factors (Additional File 2) [26,53-55].

### Domestic transmission rates

Since the contribution to the infection rate from travel-related transmission is *κ*_*i *_*λ*_*i*_, the remainder (1 - *κ*_*i*_)*λ*_*i *_is the contribution from domestic transmission. The domestic transmission rate *β*_*ij *_is the rate at which a susceptible individual of age class *i *in Canada is infected by infectious individuals of age class *j *in Canada. The resulting matrix of 7 × 7 domestic transmission rates form a WAIFW (Who Acquires Infection From Whom) matrix. By specifying a structure for the WAIFW matrix, the average values of *λ*_*i *_and *κ*_*i *_for 1980–1994 can be used along with estimated true incidence *I*_*i *_and data from seroprevalence surveys [33] to determine the domestic transmission rates β¯
 MathType@MTEF@5@5@+=feaafiart1ev1aaatCvAUfKttLearuWrP9MDH5MBPbIqV92AaeXatLxBI9gBaebbnrfifHhDYfgasaacH8akY=wiFfYdH8Gipec8Eeeu0xXdbba9frFj0=OqFfea0dXdd9vqai=hGuQ8kuc9pgc9s8qqaq=dirpe0xb9q8qiLsFr0=vr0=vr0dc8meaabaqaciaacaGaaeqabaqabeGadaaakeaaiiGacuWFYoGygaqeaaaa@2E6C@_*ij *_for 1980–1994 by solving the equation (1−κi)λi=∑j=17β¯ijIj/Nj
 MathType@MTEF@5@5@+=feaafiart1ev1aaatCvAUfKttLearuWrP9MDH5MBPbIqV92AaeXatLxBI9gBaebbnrfifHhDYfgasaacH8akY=wiFfYdH8Gipec8Eeeu0xXdbba9frFj0=OqFfea0dXdd9vqai=hGuQ8kuc9pgc9s8qqaq=dirpe0xb9q8qiLsFr0=vr0=vr0dc8meaabaqaciaacaGaaeqabaqabeGadaaakeaacqGGOaakcqaIXaqmcqGHsisliiGacqWF6oWAdaWgaaWcbaacbiGae4xAaKgabeaakiabcMcaPiab=T7aSnaaBaaaleaacqWGPbqAaeqaaOGaeyypa0ZaaabmaeaacuWFYoGygaqeamaaBaaaleaacqWGPbqAcqWGQbGAaeqaaaqaaiabdQgaQjabg2da9iabigdaXaqaaiabiEda3aqdcqGHris5aOGaemysaK0aaSbaaSqaaiabdQgaQbqabaGccqGGVaWlcqWGobGtdaWgaaWcbaGaemOAaOgabeaaaaa@48FB@ (Additional File 2).

### Modelling the cohort effect

We have estimated the average travel transmission rate τ¯
 MathType@MTEF@5@5@+=feaafiart1ev1aaatCvAUfKttLearuWrP9MDH5MBPbIqV92AaeXatLxBI9gBaebbnrfifHhDYfgasaacH8akY=wiFfYdH8Gipec8Eeeu0xXdbba9frFj0=OqFfea0dXdd9vqai=hGuQ8kuc9pgc9s8qqaq=dirpe0xb9q8qiLsFr0=vr0=vr0dc8meaabaqaciaacaGaaeqabaqabeGadaaakeaaiiGacuWFepaDgaqeaaaa@2E90@_*i *_and the average domestic transmission rate β¯
 MathType@MTEF@5@5@+=feaafiart1ev1aaatCvAUfKttLearuWrP9MDH5MBPbIqV92AaeXatLxBI9gBaebbnrfifHhDYfgasaacH8akY=wiFfYdH8Gipec8Eeeu0xXdbba9frFj0=OqFfea0dXdd9vqai=hGuQ8kuc9pgc9s8qqaq=dirpe0xb9q8qiLsFr0=vr0=vr0dc8meaabaqaciaacaGaaeqabaqabeGadaaakeaaiiGacuWFYoGygaqeaaaa@2E6C@_*ij *_for the years 1980–1994. The model could be simulated without including the cohort effect by assuming constant values *β*_*ij *_= β¯
 MathType@MTEF@5@5@+=feaafiart1ev1aaatCvAUfKttLearuWrP9MDH5MBPbIqV92AaeXatLxBI9gBaebbnrfifHhDYfgasaacH8akY=wiFfYdH8Gipec8Eeeu0xXdbba9frFj0=OqFfea0dXdd9vqai=hGuQ8kuc9pgc9s8qqaq=dirpe0xb9q8qiLsFr0=vr0=vr0dc8meaabaqaciaacaGaaeqabaqabeGadaaakeaaiiGacuWFYoGygaqeaaaa@2E6C@_*ij *_and *τ*_*i *_= τ¯
 MathType@MTEF@5@5@+=feaafiart1ev1aaatCvAUfKttLearuWrP9MDH5MBPbIqV92AaeXatLxBI9gBaebbnrfifHhDYfgasaacH8akY=wiFfYdH8Gipec8Eeeu0xXdbba9frFj0=OqFfea0dXdd9vqai=hGuQ8kuc9pgc9s8qqaq=dirpe0xb9q8qiLsFr0=vr0=vr0dc8meaabaqaciaacaGaaeqabaqabeGadaaakeaaiiGacuWFepaDgaqeaaaa@2E90@_*i *_for all time. To include the cohort effect, these constant values must be replaced by time-varying values *β*_*i *_= *β*_*i *_(*t*) and *τ*_*i *_= *τ*_*i *_(*t*).

To a good approximation, the per capita annual volume of travel by Canadian residents to HAV-endemic countries has increased quadratically since 1952, following the regression *y *= 2.38 × 10^-5 ^*t*^2 ^+ 1.52 × 10^-3 ^*t *+ 2.98 × 10^-3 ^(*R*^2 ^= 0.98) [52]. Hence, it is possible to estimate a time-varying travel transmission rate, *τ*_*i *_(*t*), by assuming that *τ*_*i *_(*t*) increased over the past half century according to the same quadratic regression, rescaled to match the known average value τ¯
 MathType@MTEF@5@5@+=feaafiart1ev1aaatCvAUfKttLearuWrP9MDH5MBPbIqV92AaeXatLxBI9gBaebbnrfifHhDYfgasaacH8akY=wiFfYdH8Gipec8Eeeu0xXdbba9frFj0=OqFfea0dXdd9vqai=hGuQ8kuc9pgc9s8qqaq=dirpe0xb9q8qiLsFr0=vr0=vr0dc8meaabaqaciaacaGaaeqabaqabeGadaaakeaaiiGacuWFepaDgaqeaaaa@2E90@_*i *_for 1980–1994 (Additional File 2).

Additionally, the domestic transmission rate *β*_*i *_(*t*) can be assumed to obey *β*_*ij *_(*t*) = β¯
 MathType@MTEF@5@5@+=feaafiart1ev1aaatCvAUfKttLearuWrP9MDH5MBPbIqV92AaeXatLxBI9gBaebbnrfifHhDYfgasaacH8akY=wiFfYdH8Gipec8Eeeu0xXdbba9frFj0=OqFfea0dXdd9vqai=hGuQ8kuc9pgc9s8qqaq=dirpe0xb9q8qiLsFr0=vr0=vr0dc8meaabaqaciaacaGaaeqabaqabeGadaaakeaaiiGacuWFYoGygaqeaaaa@2E6C@_*ij *_*F *(*t*) = β¯
 MathType@MTEF@5@5@+=feaafiart1ev1aaatCvAUfKttLearuWrP9MDH5MBPbIqV92AaeXatLxBI9gBaebbnrfifHhDYfgasaacH8akY=wiFfYdH8Gipec8Eeeu0xXdbba9frFj0=OqFfea0dXdd9vqai=hGuQ8kuc9pgc9s8qqaq=dirpe0xb9q8qiLsFr0=vr0=vr0dc8meaabaqaciaacaGaaeqabaqabeGadaaakeaaiiGacuWFYoGygaqeaaaa@2E6C@_*ij *_[*L *+ *H *(1 - tanh(*A*(*t *- *T*)))], where tanh(*t*) = (exp(2*t*) - 1)/(exp(2*t*) + 1) is the hyperbolic tangent function. The function *F*(*t*) describes a continuous decline from an upper bound to a lower bound. The parameter *L *determines the lower bound attained for sufficiently large time *t*, *L+H *determines the upper bound attained for sufficiently small *t*, *T *determines the timing of the transition between these upper and lower bounds, and *A *controls how abrupt the transition is.

We estimated *L*, *H*, *T *and *A *by exploring a plausible region of *LHTA *parameter space with fine resolution and minimizing the least-squares error between predicted seroprevalence values and the observed values from the 11 seroprevalence surveys for Canadian-born individuals of distinct age classes and survey years, between 1980 and 1994 [27,33,56-58]. It was assumed that *τ*_*i *_= *τ*_*i *_(*t*) as estimated above. With this method we obtained *L *= 0.73, *H *= 0.84, *A *= 0.038, *T *= 1960.

A previous study has also captured cohort effects by fitting a function for declining infection rates to age-stratified seroprevalence data [34]. The main differences with the previous study are that the current study includes infection due to travel in endemic countries and also explores how including the cohort effect changes the predicted impact of vaccination.

### Uncertainty analysis

To derive uncertainty intervals we applied the Latin hypercube method [59] to those parameters with the least certain values: the infection rates as estimated from catalytic modelling (*λ*_*i*_), the duration of infectiousness (*γ*_*i*_) and the proportion of travel-related incidence (*κ*_*i*_). For each sampled parameter set, the model was fitted as before to the seroprevalence data to obtain values for *L*, *H*, *T*, and *A *and then seroprevalence and incidence were computed. Details appear in Additional File 3.

## Results

### Declining transmissibility

If infection rates are assumed to be unchanged over the past century (ie, constant transmission rates β¯
 MathType@MTEF@5@5@+=feaafiart1ev1aaatCvAUfKttLearuWrP9MDH5MBPbIqV92AaeXatLxBI9gBaebbnrfifHhDYfgasaacH8akY=wiFfYdH8Gipec8Eeeu0xXdbba9frFj0=OqFfea0dXdd9vqai=hGuQ8kuc9pgc9s8qqaq=dirpe0xb9q8qiLsFr0=vr0=vr0dc8meaabaqaciaacaGaaeqabaqabeGadaaakeaaiiGacuWFYoGygaqeaaaa@2E6C@_*ij *_and τ¯
 MathType@MTEF@5@5@+=feaafiart1ev1aaatCvAUfKttLearuWrP9MDH5MBPbIqV92AaeXatLxBI9gBaebbnrfifHhDYfgasaacH8akY=wiFfYdH8Gipec8Eeeu0xXdbba9frFj0=OqFfea0dXdd9vqai=hGuQ8kuc9pgc9s8qqaq=dirpe0xb9q8qiLsFr0=vr0=vr0dc8meaabaqaciaacaGaaeqabaqabeGadaaakeaaiiGacuWFepaDgaqeaaaa@2E90@_*i *_are assumed) then the modelled seroprevalence profile exhibits a large error relative to the observed data from the 11 seroprevalence surveys for Canadian-born individuals (Table 1) [27,33,56-58]. The predicted seroprevalence is too large in the youngest age classes and too small in the oldest age classes. The predicted incidence is significantly larger than observed incidence for all age classes (Table 2).

**Table 1:** Observed seroprevalence versus fitted seroprevalence for the the case of constant domestic (*β*_*ij *_= β¯
 MathType@MTEF@5@5@+=feaafiart1ev1aaatCvAUfKttLearuWrP9MDH5MBPbIqV92AaeXatLxBI9gBaebbnrfifHhDYfgasaacH8akY=wiFfYdH8Gipec8Eeeu0xXdbba9frFj0=OqFfea0dXdd9vqai=hGuQ8kuc9pgc9s8qqaq=dirpe0xb9q8qiLsFr0=vr0=vr0dc8meaabaqaciaacaGaaeqabaqabeGadaaakeaaiiGacuWFYoGygaqeaaaa@2E6C@_*ij*_) and travel (*τ*_*i *_= τ¯
 MathType@MTEF@5@5@+=feaafiart1ev1aaatCvAUfKttLearuWrP9MDH5MBPbIqV92AaeXatLxBI9gBaebbnrfifHhDYfgasaacH8akY=wiFfYdH8Gipec8Eeeu0xXdbba9frFj0=OqFfea0dXdd9vqai=hGuQ8kuc9pgc9s8qqaq=dirpe0xb9q8qiLsFr0=vr0=vr0dc8meaabaqaciaacaGaaeqabaqabeGadaaakeaaiiGacuWFepaDgaqeaaaa@2E90@_*i*_) transmission rates, and for the case of time-varying domestic (*β*_*ij *_= *β*_*ij *_(*t*)) and travel (*τ*_*i *_= *τ*_*i *_(*t*)) transmission rates.

	Seroprevalence		
	
Age class, year, survey	Observed	Fitted, without cohort effect	Fitted, with cohort effect
0–4, 1981, Ref. 58	0.0024	0.014	0.009
5–9, 1981, Ref. 58	0.0024	0.047	0.030
10–19, 1988, Ref. 59	0.0063	0.068	0.046
10–19, 1995, Ref. 27	0.0299	0.068	0.048
10–19, 1981, Ref. 57	0.0632	0.068	0.049
20–29, 1988, Ref. 59	0.103	0.082	0.064
20–29, 1980, Ref. 57	0.114	0.082	0.129
30–39, 1988, Ref. 59	0.287	0.097	0.171
40–59, 1980, Ref. 57	0.599	0.115	0.618
40–59, 1988, Ref. 59	0.550	0.115	0.491
60+, 1988, Ref. 59	0.820	0.137	0.745

The seroprevalence values listed under "Observed", "Without cohort effect" (fitted model, constant case) and "With cohort effect" (fitted model, time-varying case) are for the timepoints specified in the first column.

**Table 2:** Average observed incidence of reported cases (per 100,000 per year) versus predicted incidence of reported cases for the case of constant domestic (*β*_*ij *_= β¯
 MathType@MTEF@5@5@+=feaafiart1ev1aaatCvAUfKttLearuWrP9MDH5MBPbIqV92AaeXatLxBI9gBaebbnrfifHhDYfgasaacH8akY=wiFfYdH8Gipec8Eeeu0xXdbba9frFj0=OqFfea0dXdd9vqai=hGuQ8kuc9pgc9s8qqaq=dirpe0xb9q8qiLsFr0=vr0=vr0dc8meaabaqaciaacaGaaeqabaqabeGadaaakeaaiiGacuWFYoGygaqeaaaa@2E6C@_*ij*_) and travel (*τ*_*i *_= τ¯
 MathType@MTEF@5@5@+=feaafiart1ev1aaatCvAUfKttLearuWrP9MDH5MBPbIqV92AaeXatLxBI9gBaebbnrfifHhDYfgasaacH8akY=wiFfYdH8Gipec8Eeeu0xXdbba9frFj0=OqFfea0dXdd9vqai=hGuQ8kuc9pgc9s8qqaq=dirpe0xb9q8qiLsFr0=vr0=vr0dc8meaabaqaciaacaGaaeqabaqabeGadaaakeaaiiGacuWFepaDgaqeaaaa@2E90@_*i*_) transmission rates, and for the case of time-varying domestic (*β*_*ij *_= *β*_*ij *_(*t*)) and travel (*τ*_*i *_= *τ*_*i *_(*t*)) transmission rates, from 1980–1994.

	Average incidence of reported cases, 1980–1994		
	
Age Class	Observed	Predicted, without cohort effect	Predicted, with cohort effect
0–4	6.8	10.3 (1.4, 19.2)	7.4 (5.7, 11.6)
5–9	17.0	21.8 (3.3, 40.3)	15.7 (11.9, 23.9)
10–19	7.8	13.8 (4.6, 23.0)	9.6 (7.4, 12.1)
20–29	9.4	14.8 (2.8, 26.8)	9.8 (7.3, 13.8)
30–39	7.2	16.1 (1.4, 30.8)	9.4 (7.0, 13.4)
40–59	3.7	11.3 (1.9, 20.7)	4.0 (3.1, 5.3)
60+	1.8	12.2 (0.8, 23.6)	2.2 (1.6, 3.2)
All ages	6.5	13.8 (2.2, 25.4)	7.5 (5.2, 10.7)

The quantities in brackets represent 96% uncertainty intervals (see Methods).

By comparison, there is good agreement between fitted and observed seroprevalence when infection rates *β*_*ij *_(*t*) and *τ*_*i *_(*t*) evolve over time as described in Methods (Table 1). Accounting for the cohort effect through a declining transmissibility function explains most of the difference between fitted and observed seroprevalence (*R*^2 ^= 0.97). The remaining discrepancies may partly be due to other heterogeneities not accounted for, such as outbreaks in high risk groups during specific periods, or constraints inherent to the hyperbolic tangent model. The fitted seroprevalence remains somewhat high in the younger age classes and somewhat low in the older age classes. The agreement between predicted and observed incidence also improves dramatically once the cohort effect is included (Table 2). Observed incidence values fall within 96% uncertainty intervals of the predicted values. A piecewise exponential function for *F*(*t*) yields similar results.

**Figure 4:**
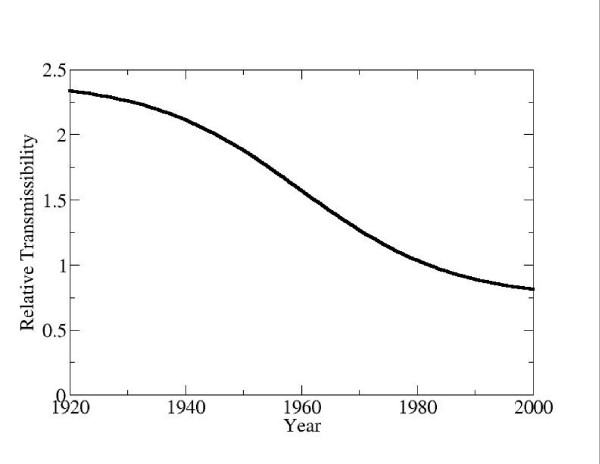
**HAV transmissibility over time, as determined from fitting the dynamic model to seroprevalence data**. The vertical axis shows the function *F*(t) which describes time evolution of transmissibility.

Figure 4 illustrates the time evolution of transmissibility *F*(*t*) over the past century from the fitted model. Transmissibility has dropped by a factor of 2.8 since the 1920s. The period of most rapid change was from 1950 to 1970, which is consistent with an era of rapid improvements in hygiene and sanitation in Canada, due ultimately to post-war economic growth.

### Universal vaccination

Here we compare the predicted effects of universal vaccination in Canada over 20-year and 50-year periods starting in 2006, with and without inclusion of the cohort effect in the dynamic model. For the case of constant transmissibility (cohort effect is not included), transmission rates in the vaccine era are assumed to be constant (*β*_*ij *_= β¯
 MathType@MTEF@5@5@+=feaafiart1ev1aaatCvAUfKttLearuWrP9MDH5MBPbIqV92AaeXatLxBI9gBaebbnrfifHhDYfgasaacH8akY=wiFfYdH8Gipec8Eeeu0xXdbba9frFj0=OqFfea0dXdd9vqai=hGuQ8kuc9pgc9s8qqaq=dirpe0xb9q8qiLsFr0=vr0=vr0dc8meaabaqaciaacaGaaeqabaqabeGadaaakeaaiiGacuWFYoGygaqeaaaa@2E6C@_*ij *_*τ*_*i *_= τ¯
 MathType@MTEF@5@5@+=feaafiart1ev1aaatCvAUfKttLearuWrP9MDH5MBPbIqV92AaeXatLxBI9gBaebbnrfifHhDYfgasaacH8akY=wiFfYdH8Gipec8Eeeu0xXdbba9frFj0=OqFfea0dXdd9vqai=hGuQ8kuc9pgc9s8qqaq=dirpe0xb9q8qiLsFr0=vr0=vr0dc8meaabaqaciaacaGaaeqabaqabeGadaaakeaaiiGacuWFepaDgaqeaaaa@2E90@_*i*_, as in Methods). For the case of declining transmissibility (cohort effect is included), transmission rates in the vaccine era are given by *β*_*ij *_= *β*_*ij *_(*t*), *τ*_*i *_= *τ*_*i *_(*t*), the same functions used to fit the model to the seroprevalence surveys but extrapolated to the vaccine era. The effects of the targeted policy in place from 1995 to the present are accounted for by reducing the travel transmission rate until the reported age-specific incidence from 1995–2005 is recovered, and thereafter the targeted policy is discontinued. Other parameters are unchanged from 1980–1994 values. Vaccination is applied to 4-year-olds and calls for two doses of vaccine with 70% compliance and 97% vaccine efficacy [49,50]. Vaccination occurs at age 4 because this would allow Hepatitis A vaccination to be efficiently scheduled along with other currently-administered vaccines in Canada. The assumed rate of waning of vaccine-derived immunity was 1.65% per year [47], which is a conservative assumption since cell-mediated immunity may actually result in lifetime protection.

Over both 20- and 50-year periods, failure to include the cohort effect over-estimates incidence and mortality, both with and without vaccination (Tables 3, 4). If the cohort effect is not included, the average reported incidence over 20 years is predicted to be 7.8 per 100,000 per year. If the cohort effect is included, it is 5.8 per 100,000 per year. Likewise, the cumulative number of deaths attributable to HAV over 20 years is predicted to be 262 instead of 138. Hence, from the perspective of absolute incidence in the vaccine era, not including the cohort effect will under-estimate the effectiveness of vaccination.

**Table 3:** Predicted annual incidence of reported cases, with and without including the cohort effect in the dynamic model.

	Average annual reported incidence (cases per 100,000 per year)							
	
	Over 20 years (2006–2025)				Over 50 years (2006–2055)			
	
	Without cohort effect		With cohort effect		Without cohort effect		With cohort effect	
	
Age Class	Vaccination	No vaccination	Vaccination	No vaccination	Vaccination	No vaccination	Vaccination	No vaccination
0–4	6.4	10.3	5.6	7.9	6.2	10.3	5.4	7.6
5–9	7.0	21.8	6.2	16.9	5.8	21.8	5.1	16.3
10–19	6.9	13.8	5.9	10.3	5.0	13.8	4.3	10.0
20–29	9.4	14.8	7.8	10.6	6.6	14.8	5.5	10.2
30–39	10.1	16.1	8.3	11.3	8.1	16.1	6.5	10.8
40–59	7.0	11.3	4.8	6.7	6.3	11.3	4.7	7.0
60+	7.3	12.2	2.7	3.9	6.8	12.2	3.8	5.7
All Ages	7.8	13.8	5.8	9.0	6.4	13.8	5.0	9.1

**Table 4:** Predicted cumulative number of deaths attributable to HAV, with and without including the cohort effect in the dynamic model.

	Cumulative number of deaths							
	
	Over 20 years (2006–2025)				Over 50 years (2006–2055)			
	
	Without cohort effect		With cohort effect		Without cohort effect		With cohort effect	
	
Age Class	Vaccination	No vaccination	Vaccination	No vaccination	Vaccination	No vaccination	Vaccination	No vaccination
0–4	7.6	12.3	6.8	9.5	18.5	30.8	16.2	22.9
5–9	5.0	15.7	4.4	12.1	10.5	39.2	9.2	29.3
10–19	9.9	19.9	8.5	14.9	17.9	49.6	15.5	36.0
20–29	13.5	21.3	11.3	15.3	23.7	53.2	19.7	36.6
30–39	17.0	27.1	13.9	19.0	33.9	67.7	27.5	45.5
40–59	48.9	79.2	33.6	46.8	109.7	198.0	81.7	122.7
60+	159.6	265.6	59.3	85.6	367.7	664.0	205.5	311.4
All Ages	261.6	441.0	137.9	203.2	581.8	1102.5	375.3	604.5

On the other hand, from the perspective of percentage reductions in incidence and mortality brought on by vaccination, the effectiveness of vaccination can be significantly over-estimated if the cohort effect is not included. In this case the percentage reduction in incidence (respectively mortality) due to vaccination is 43% (respectively 83%) over a 20-year period if the cohort effect is not included. These figures are 23% (respectively 73%) when the cohort effect is included. Patterns are similar over 50 years. The drop in mortality brought on by vaccination is largest in the oldest age classes since older individuals tend to be at a higher risk for mortality attributable to HAV.

The absolute and relative differences between incidence and mortality with and without vaccination, over 20-year and 50-year periods, are also larger when the cohort effect is not included.

We note that the average annual reported cases and deaths are somewhat higher over the 50-year period than the 20-year period (Tables 3 and 4). This occurs because the travel transmission rate after 2006 continues to climb according to our fitted function *τ*_*i *_= *τ*_*i *_(*t*) that reflects long-term historical trends in travel. Hence, the number of individuals infected through travel to HAV-endemic countries continues to rise, while the fall in the domestic transmission rate *β*_*ij *_= *β*_*ij *_(*t*) after 2006 is much more moderate. Another possible reason for this effect is that a decrease in incidence in the younger, recently-vaccinated age classes is offset by an increase in incidence in the older age classes, who are increasingly susceptible as more time passes since the cessation of the (all-ages) targeted policy in 2006. We have assumed that vaccine-derived immunity wanes, and so not all individuals vaccinated as children retain their immunity when older. Older age classes also experience higher rates of mortality attributable to HAV, so the net number of deaths can increase over time.

## Discussion

These results indicate that the presence of a cohort effect can significantly alter the impact of vaccination. The implicit assumption that infection rates have always been constant is frequently made in modelling studies, but it is incorrect for many common infectious diseases. Failure to include the cohort effect overestimates incidence and mortality both before and after introduction of universal vaccination, as well as the percentage reduction achieved by universal vaccination.

Cohort effects are not immediately obvious in case reporting data or seroprevalence survey data. In seroprevalence data, it is often difficult to disentangle the two dimensions – calendar year and age – that are present in any set of seroprevalence surveys (Figure 2). In case reporting data, there is not a simple one-to-one relationship between reported incidence and true infection rates, particularly in cases where the probability of clinically apparent infection varies with age. In some situations, a rise in reported incidence may actually signal reduced infection rates in the general population or in certain age cohorts. This may have happened in Canada in the last decades of the twentieth century. Reported incidence in Hepatitis A in Canada exhibits an increasing trend between the years of 1980 and 1996 (linear regression: y = 0.21x + 4.61, *R*^2 ^= 0.24; the *R*^2^ value is low because of the recurrent outbreaks). The dynamic model during this time predicts an increase in reported incidence (linear regression: y = 0.18x + 5.92, *R*^2 ^= 0.99) while simultaneously predicting a decrease in true (actual) incidence due to a declining transmission rate (Figure 4). This occurs because declining transmissibility yields a decrease in the infection rates and hence an increase in the mean age at infection. Because of age dependence in the probability of clinical infection, this translates into more reported HAV infections. However, in other situations, variation in reported incidence over time may also be due to changing case reporting methods.

Because of such subtleties, dynamic modelling provides a rigorous and transparent framework for the interpretation and synthesis of seroprevalence and case reporting, and especially the clarification of cohort effects (eg, Figure 4). Modelling is valuable not only for predicting the impact of proposed policies but also for understanding and interpreting epidemiologic data. When developing dynamic models of disease transmission and vaccination, seroprevalence data should be interpreted carefully and incorporated into model parameterization whenever possible.

In our parameterization, some of the seroprevalence survey data were used twice. Firstly, they were used in the catalytic modelling to determine the proportion of seropositive individuals and hence the infection rates. Secondly, they were used to fit the function *F*(t) for declining transmissibility. This two-step approach may introduce bias, since it assumes transmissibility has declined in each age class in the same way over the past century. An alternative approach might have been to fit functions for declining transmissibility for each age class separately. However, this would mean estimating 10 parameters using 11 data points, which constitutes a poorly determined problem. In situations where more data points are available, this latter approach could be desirable.

In Canada, Hepatitis A transmission is primarily person-to-person. For other countries where environmental transmission is a significant source of Hepatitis A infection, the model would have to be modified to take environmental transmission into account [60].

Although this study addresses HAV specifically, the results should apply to any infectious disease in which there is lifelong immunity, person-to-person transmission, and where infection rates have changed significantly in past decades. Many pediatric infectious diseases fall into this category, with one difference being that disease severity declines with age instead of increasing, as with HAV. Hence, for many dynamic modelling studies, it may be necessary to account for cohort effects.

## Conclusion

Failure to account for cohort effects has implications for interpreting seroprevalence survey data and parameterizing dynamic models. Moreover, for Hepatitis A, the predicted impact of universal vaccination can vary widely depending on whether the cohort effect is included in the dynamic model. This is likely to be true for any infectious disease with lifelong immunity in populations with pronounced cohort effects. Hence, cohort effects should be accounted for in dynamic modelling studies whenever applicable.

## Competing interests

This study was funded by a Canadian Institutes of Health Research Industry-Partnered Operating Grant in connection with GlaxoSmithKline (GSK). BZP and ACT were employed by GSK. CTB was a consultant for GSK. CTB, BD and VG have received research funding from GSK.

## Authors' contributions

All authors provided feedback on the modelling and the writing of manuscript as well as background to mathematical, epidemiological, clinical and/or public health literature. ASRSR programmed and helped construct the dynamic model, and wrote early drafts of the manuscript; MHC conducted the catalytic modelling under supervision of BZP; ACT reviewed the literature on Hepatitis A epidemiology; VG, BD and MDK provided guidance and feedback on epidemiological and public health aspects of HAV transmission and vaccination; CTB conceived of the study, constructed and analyzed the dynamic model, and completed the writing of the manuscript. All authors read and approved the final manuscript.

## Pre-publication history

The pre-publication history for this paper can be accessed here:



## Supplementary Material

Additional File 1 **Model Equations**. This file defines the model equations, parameters and variables. Click here for file

Additional File 2 **Parameter Estimation**. This file describes the model parameterization. Click here for file

Additional File 3 **Uncertainty Analysis**. This file describes the methods of uncertainty analysis. Click here for file
