# Supplementary material for: Cohort effects in dynamic models and their impact on vaccination programmes: an example from Hepatitis A
Source: BMC Infect Dis. 2006 Dec 5;6:174. doi: 10.1186/1471-2334-6-174 (PMC1702544; doi:10.1186/1471-2334-6-174)
Supplement: Additional File 1 — Model Equations. This file defines the model equations, parameters and variables. [file 1471-2334-6-174-S1.pdf]

Additional File 1. Model Equations

The ordinary differential equations that govern the time evolution of state variables (S_i, E_i, I_i, R_i, V_i) during each year are:

$$
\begin{aligned}
\frac{dS_i}{dt} &= -S_i \sum_{j=1}^{7} \beta_{ij} I_j / N_j - \tau_i S_i - g_i + fV_i \\
\frac{dE_i}{dt} &= S_i \sum_{j=1}^{7} \beta_{ij} I_j / N_j + \tau_i S_i - \delta E_i \\
\frac{dI_i}{dt} &= -\gamma_i I_i + \delta E_i \\
\frac{dR_i}{dt} &= \gamma_i I_i \\
\frac{dV_i}{dt} &= g_i - fV_i
\end{aligned}
\qquad \text{(A1)}
$$

where f is the mean rate at which vaccinated individuals lose their immunity (equivalently, $1/f$ is the mean duration of vaccine-derived immunity); δ is the mean rate at which exposed individuals become infectious (equivalently, $1/\delta$ is mean latent period); γ_i is the rate at which infected individuals in age class *i* recover to a state of lifelong immunity (equivalently, $1/\gamma_i$ is mean infectious period for individuals in age class *i*); ε is the vaccine efficacy; g_i is the rate at which individuals in age class *i* are vaccinated; β_{ij} is the rate at which a susceptible in age class *i* becomes infected by contact with infectious individuals in age class *j* in Canada, and τ_i is the rate at which a susceptible in age class *i* becomes infected through contact with infectious individuals while travelling overseas. At the end of each year, individuals of a given age *y* (where *y*, an integer, is the age in years) are moved to the next age *y*+1. If *y* = 4, 9, 19, 29, 39, or 59, they will also move into the next highest age class. Each year, new individuals are born susceptible in the lowest age, *y*=0.
