# Supplementary material for: Cohort effects in dynamic models and their impact on vaccination programmes: an example from Hepatitis A
Source: BMC Infect Dis. 2006 Dec 5;6:174. doi: 10.1186/1471-2334-6-174 (PMC1702544; doi:10.1186/1471-2334-6-174)
Supplement: Additional File 2 — Parameter Estimation. This file describes the model parameterization. [file 1471-2334-6-174-S2.pdf]

Additional File 2. Parameter Estimation

The model was parameterized using data from 1980-1994. Incidence and seroprevalence data for Canada are both available after 1980, and 1994 marks the year of vaccine licensure in Canada and thus a change in transmission patterns. Table A1 summarizes parameter values and relevant literature sources (see main text for references, except for references 61-65, which appear at the end of this file). Parameterization is discussed in greater detail below.

Table A1: Parameter values used in dynamic model, with literature sources.

Parameter	Meaning	Value Used	Relevant References
$\lambda_1 \ldots \lambda_7$ (used to estimate β_{ij}, τ_i)	age-specific force of infection	689.7, 488.7,115.9, 129.0, 156.4, 97.7, 117.9 per 100000 per year	[14, 27, 33, 56-58], Online Appendix 2
$\kappa_1 \ldots \kappa_7$ (used to estimate β_{ij}, τ_i)	age-specific proportion of cases attributable to travel	0.37, 0.37, 0.37, 0.27, 0.22, 0.20, 0.15	[26,52-54], Online Appendix 2
ρ	Under-reporting	7.6	[14,32,33] Online Appendix 2
B	birth rate	400000	[51]
$d_1 \ldots d_7$	age-specific non-HAV death rate	0, 0, 0, 0, 0.75, 0.67, 1	[51]
$N_1 \ldots N_7$	population sizes of age classes	2, 2, 4, 4, 4, 6, 4 (millions)	[51]
$1/\delta$	mean duration of latent	2 weeks	[44,45]

	period		
ε	Vaccine efficacy	0.97	[49,50]
F	Rate of loss of vaccine-derived immunity	0.0165 $year^{-1}$	[47]
$1/\gamma_1 ... 1/\gamma_7$	age-specific mean duration of infectious period	3.5, 3.0, 2.5, 2.5, 2.5, 2.5, 2.5 weeks	[45,46]
$\theta_1 ... \theta_7$	age-specific death rate in symptomatic individuals due to HAV infection	30, 18, 18, 18, 21, 58.5, 272 per 10,000	[47,48]

Domestic transmission rate β_{ij} and travel transmission rate τ_i

The transmission rates β_{ij} and τ_i were obtained from the force of infection λ_i, the proportion of cases due to travel in endemic countries κ_i [26,53-55], and data on volume of travel to endemic countries over time [52]. The quantities λ_i and κ_i in turn were estimated by integrating incidence data [14] and seroprevalence data [27, 33, 56-58] through catalytic modelling [31,32].

Catalytic modelling involves fitting a model for the age-specific probability of jaundice and a model for the force of infection as a function of time and age class. It is assumed that the force of infection can be broken down into separate age-related and time-related components. Incidence data are reconciled with seroprevalence data by adjusting for the probability of subclinical infection and for under-reporting via least-squares fitting to the jaundice and force of infection models. The catalytic modelling procedure used for this study was identical to that of a previous study for HAV [31] except that a piecewise linear jaundice model was used for the present study instead: $P_J(A) = \beta A$ (for $A<\alpha$) and $P_J(A) = \beta\alpha$ (for $A\geq\alpha$) (where $P_J(A)$ is the probability of developing jaundice at age A, and where β, α are free parameters that are fitted from the data). A piecewise linear model was found to yield estimates of true incidence that were more consistent with the observed seroprevalence. The piecewise linear jaundice model was fitted to age-specific

data on the probability of jaundice from seven studies [6,15-19,31], weighted according to the sample size of the study. The probabilities of jaundice thus computed were, for the seven age classes in order: 0.11, 0.34, 0.70, 0.81, 0.81, 0.81, 0.81. The under-reporting factor was computed to be 7.6 (Pham et al, unbpublished data, 2006). To capture the effects of targeted vaccination from 1995 to 2005, the travel transmission rate was reduced such that the average incidence from 1995 to 2005 was recovered.

The average true incidence I_j for 1980-1994 was estimated by adjusting the average reported incidence from 1980-1994 for jaundice and under-reporting. The force of infection λ_i in turn was estimated by dividing I_j by the proportion of individuals susceptible in age class i, as determined from the seroprevalence surveys [33]. Since the total force of infection is $\lambda_i = \tau_i + \sum_{j=1}^{7} \beta_{ij} I_j / N_j$, and since $\tau_i = \lambda_i \kappa_i$, we have that $\lambda_i (1 - \kappa_i) = \sum_{j=1}^{7} \beta_{ij} I_j / N_j$. With the values of λ_i and I_j estimated as above and the values for κ_i known [26,52-54], it is possible in principle to solve for β_{ij}. The set of 49 values for β_{ij} form a "Who Acquires Infection From Whom" matrix. However, because there are more unknown matrix entries (49) than equations (7), it is necessary to assume that each WAIFW matrix entry can take on only one of 7 distinct values, denoted ω_1 through ω_7.

We assumed that $\beta_{11}=\overline{\omega}_1$, $\beta_{12}=\beta_{22}=\beta_{21}=\overline{\omega}_2$, $\beta_{13}=\beta_{23}=\beta_{33}=\beta_{32}=\beta_{31}=\overline{\omega}_3$, etc (Table A2). After experimenting with various types of WAIFW matrices [39], we found that this WAIFW matrix gave good agreement with seroprevalence data, while also being the most robust to changes in parameter values (this robustness is needed for uncertainty analysis, Online Appendix 3). Hence, this WAIFW matrix was used throughout. Other forms of the WAIFW matrix, such as those incorporating specific entries for parent-child transmission, assymmetric transmission between parents and children, or elements of assortative mixing, were found to be less robust to changes in parameters and/or gave less accurate fits to the seroprevalence data. The form used here is similar to forms that have been used for other viral diseases spread by person-to-person contact and in which childhood transmission is very important, such as measles [39].

The values of $\overline{\omega}_i$ obtained by solving the equation $\sum_{j=1}^{7} \beta_{ij} I_j / N_j = (1-\kappa_i)\lambda_i$ using the WAIFW matrix in Table A2 are $\overline{\omega}_{1..7}$= 7.33, 4.30, 0.81, 1.06, 1.39, 0.87, and 1.11 yr^{-1}. Transmission rates are largest in the youngest age classes, and generally decrease along the diagonal of the WAIFW matrix as one moves in the direction of older age classes, except for a noticeable increase in the 30-39 age class. The transmission rates also decrease in the direction of the cross-diagonal, ie, in the direction of greater disassortative mixing. This pattern is observed for WAIFW matrices for many infectious diseases [61].

The parameter κ_i is the proportion of Hepatitis A cases presenting in Canada that were acquired by Canadian residents travelling in endemic countries in age class *i* [25,52-55]. We scaled vaccine-era age specific data to match pre-vaccine era non-age-structured data to obtain the following age-specific values for κ_i from 1980-1994: 37% (0-4, 5-9, 10-19), 27% (20-29), 22% (30-39), 20% (40-59), 14% (60+). The values of $\overline{\tau}_i$ were then computed from $\overline{\tau}_i = \lambda_i \kappa_i$.

Table A2: WAIFW Matrix used in the dynamic model.

	Index case							
	Age Class	0-4	5-9	10-19	20-29	30-39	40-59	60+
Infected case	0-4	ω_1	ω_2	ω_3	ω_4	ω_5	ω_6	ω_7
	5-9	ω_2	ω_2	ω_3	ω_4	ω_5	ω_6	ω_7
	10-19	ω_3	ω_3	ω_3	ω_4	ω_5	ω_6	ω_7
	20-29	ω_4	ω_4	ω_4	ω_4	ω_5	ω_6	ω_7
	30-39	ω_5	ω_5	ω_5	ω_5	ω_5	ω_6	ω_7
	40-59	ω_6	ω_6	ω_6	ω_6	ω_6	ω_6	ω_7
	60+	ω_7	ω_7	ω_7	ω_7	ω_7	ω_7	ω_7
Who Acquires Infection from Whom Matrix								

Total number of births per year (*b*): Each year we assume that 400,000 individuals are born. This value is close to the average annual number of births in Canada from 1980-1994 [51].

Population sizes (N_i): We took the sizes of the age classes to be N_1=2, N_2=2, N_3=4, N_4=4, N_5=4, N_6=6, and N_7=4 (in millions). These estimates are close to the average sizes for 1980-1994 [51].

Death rates (d_i): The death rate in Canada from 1980 to 1996 is very small in all age classes except for 40-59 and 60+ [51]. The sizes of age classes reflect both age-specific mortality rates and demographic factors (e.g. the 'baby boom'). To reproduce the average population sizes of the age classes between 1980 and 1996, and to take into account the increased death rate in older age classes, we assumed a proportion 0.75 of individuals die upon entering the 40-59 age class, a proportion 0.67 die upon entering the 60+ age class, and no individual lives more than 80 years. With these birth and death rules, the total population size and the size of each age class remained constant. Although population size has actually increased over the past century, assuming a fixed population will not affect predictions of measures that indicate the proportion of individuals subject to a certain condition, such as incidence rates or mortality rates. This is because we use "standard" incidence function for dynamic modelling (incidence $=\sum \beta_{ij} I_j / N_j$) [62] instead of the more traditional but less accurate "mass-action" incidence function (incidence = $\sum \beta_{ij} I_j$). For standard incidence, the force of infection is proportional to the percentage infected, not the absolute number. Hence, a gradually increasing population size will not affect the force of infection, so long as population density (and thus the average number of social contacts of a susceptible individual) is constant.

Rate of loss of immunity (*f*): *f* is the rate at which a vaccinated individual loses vaccine-derived immunity. By fitting an exponential function to a waning immunity curve

arrived at by expert consensus [47], we estimate an annual rate of decline of 1.65% for vaccine-derived immunity from a two-dose course.

Vaccine efficacy (ε): ε is the vaccine efficacy (proportion of vaccinated individuals who thereby obtain immunity against infection). We estimate the efficacy of a two-dose course to be $\varepsilon = 0.97$, based on the average result from two different randomized trials [49,50].

Mean latent period ($1/\delta$): δ is the mean rate at which an exposed individual becomes infectious, and is the inverse of the mean latent period. The average incubation period for HAV is 28 days [44], and the average time between the start of viral shedding and the onset of symptoms is 14 days [45], so the average latent period, which is the time from infection to the onset of viral shedding, is 28-14 days = 14 days. Hence we take δ = 365/14 = 26.07 year^{-1}. There does not appear to be significant age-related variation in the latent period.

Mean effective infectious period ($1/\gamma_i$): γ_i is the average rate at which an infected individual moves from the infectious compartment to the removed compartment, and hence the inverse $1/\gamma_i$ is the mean duration of infectiousness. HAV is shed from one to three weeks before the onset of symptoms and continues to be shed for a week or more after the onset of symptoms [46], and children shed the virus longer than adults [45]. The duration of viral shedding can vary from between a few days to 6 months in infants [63] and up to 3 months in older children and adults [64]. However, data from epidemiological studies suggest that peak infectivity occurs during the two weeks before the onset of symptoms and the week after jaundice appears [7, 65]. However, epidemiologic data suggest that relatively few transmission events occurs after the onset of symptoms (with or without exclusion), despite the continued shedding of the virus. Hence we assume the mean effective infectious period was 3.5 weeks in the 0-4 age class, 3.0 weeks in the 5-9 age class, and 2.5 weeks for all other age classes. This yields γ_1 = 14.9 year $^{-1}$, γ_2 = 17.4 year $^{-1}$, and $\gamma_3 = \gamma_4 = \gamma_5 = \gamma_6 = \gamma_7$ = 20.9 year $^{-1}$.

Age-specific mortality rates: We based our parameter values for mortality rates attributable to HAV in symptomatic individuals on previous estimates (per 10,000): 30 in 0-4 [47,48], 18 in 15-19, 18 in 20-29, 21 in 30-39, 36 in 40-49, 81 in 50-59, 149 in 60-69, 283 in 70-79, and 385 in 80+. We averaged these estimates for the 40-59 and 60+ age classes and assumed the 15-19 rate applied to the 10-19 age class. We adjusted for the probability of jaundice to obtain age-specific mortality rates per infected case.

Additional References for Additional File 2:

61. Farrington CP and Whitaker HJ: **Contact surface models for infectious diseases: estimation from serologic survey data.** *J Am Stat Assoc* 2005, **100**:370-379.
62. Hethcote HW, Wang W and Li Y: **Species coexistence and periodicity in host-host-pathogen models.** *J Math Biol* 2005, **51:** 629-660.
63. Rosenblum LS, Villarino LM, Nainan OV et al: **Hepatitis outbreak in neonatal intensive care unit: risk factors for transmission and evidence of prolonged viral excretion among preterm infants**. *J Inf Dis* 1991, **164**(**3**):33476-33482.
64. Bower WA, Nainan OV, Han X et al: **Duration of viremia in hepatitis A virus infection**. *J Infect Dis* 2000, **182**:12-17.
65. Richardson M, Elliman D, Maguire H et al: **Evidence base of incubation periods, periods of infectiousness and exclusion policies for the control of communicable disease in schools and preschools.** *Ped Inf Dis J* 2001, **20**:380-391.
