# Supplementary material for: Cohort effects in dynamic models and their impact on vaccination programmes: an example from Hepatitis A
Source: BMC Infect Dis. 2006 Dec 5;6:174. doi: 10.1186/1471-2334-6-174 (PMC1702544; doi:10.1186/1471-2334-6-174)
Supplement: Additional File 3 — Uncertainty Analysis. This file describes the methods of uncertainty analysis. [file 1471-2334-6-174-S3.pdf]

Additional File 3. Uncertainty analysis

For uncertainty analysis, bounds of uncertainty were selected for three sets of parameters: the age-specific forces of infection as estimated from catalytic modelling (λ_i), the age-specific duration of infectiousness (γ_i) and the age-specific proportion of travel-related incidence (κ_i).

We assumed that all errors in the 'best guess' parameter values for these three sets of parameters would be systematic across age classes. For instance, if the 'best guess' duration of infectiousness is too high in the 0-4 age class, it will also be too high in other age classes. This amounts to claiming that, in the uncertainty analysis, it should not be reasonable to test a set of parameter values where the duration of infectiousness is two weeks in the 0-4 age class and (a much higher) four weeks in the 5-9 age class. Hence, the deviations from the best guess parameter values were applied uniformly across age classes; for instance, we investigate the effect of scaling γ_i by some constant *c* for all *i*.

Forces of infection: To select upper and lower bounds of uncertainty for the age-specific force of infection (λ_i^{upper} and λ_i^{lower} respectively), we scaled the best guess force of infection values (λ_i) predicted from the catalytic modelling: each force of infection value λ_i was scaled upward or downward by the same constant α to obtain upper and lower bounds.

For the upper bound ($\alpha > 1$), we used the force of infection values as computed from the same catalytic modelling algorithm as before but using a different jaundice model: $P_J(a) = P_J^{MAX}(1-\exp(-ra^s))$ [31]. This jaundice model has been used by others [31] and predicts a lower probability of jaundice (and hence higher force of infection) than the piecewise linear model used in this paper. λ_1^{upper} was set to be the value for the force of infection computed from this alternative jaundice model for the lowest age class, *i*=1. Since λ_1^{upper}=0.001461 and λ_1=0.000690, this yields α = 0.001461/0.000690 = 2.12. The

upper bound λ_i^{upper} for the remaining age classes, i=2...7, were then computed as $\lambda_i^{\text{upper}} = 2.12\lambda_i$.

To come up with a lower bound ($\alpha < 1$), we compared the predicted seroprevalence from the catalytic modelling to the observed seroprevalence in the third age class (10-19) from several seroprevalence studies, and adjusted α so that the predicted seroprevalence matched the observed seroprevalence. The third age class was chosen because (1) most individuals in this age class lived through most of 1980-1996, the time for which force of infection estimates were made, and (2) there are three independent seroprevalence studies for this age class, for the years 1980, 1988 and 1996 [27,33,56-58], as opposed to only one for 0-9 [57]. The average seroprevalence from these three surveys was 0.033. The predicted seroprevalence (S_i) in age class i can be determined from the estimated forces of infection (λ_i) via

$$S_i = \exp(-\lambda_i T_i/2) + \sum_{j=1}^{i-1} \exp(-\lambda_j T_j), \qquad \text{(A2)}$$

where T_i is the width, in years, of age class i. The lower bound was chosen as the value of the rescaling parameter α for which the seroprevalence in the third age class predicted from catalytic modelling agreed with the observed seroprevalence, 0.033. This value was $\alpha = 0.52$.

Duration of infectiousness: Despite the fact that viral shedding continues after the onset of symptoms [45], epidemiologic data imply that transmission events are relatively rare beyond this point [65; see end of file for reference]. Therefore, the lower range for the duration of infectiousness was taken to be two weeks (which is the time between the end of the latent period and the onset of symptoms) for all age classes, and the upper ranges were taken to be four weeks for age classes 0-4 and 5-9 and three weeks in all other age classes.

Travel-related incidence: The study of Hepatitis A risk factors conducted in Montreal during the years 1993-1995 (before the vaccine era) estimated that 26.3% of infections reported in Montreal originated during travel by Canadian residents overseas [53]. One

potential risk in extrapolating this study to the rest of Canada is that Montreal may not be representative. However, more recent studies (in the vaccine era) across the four largest Canadian provinces show that the proportion of travel-related incidence is similar in these provinces [55], hence this extrapolation is reasonable. Nonetheless, the Montreal estimates may be biased high, since Montreal is an urban centre and a higher proportion of individuals who live in urban centres travel overseas. (The four provinces studied more recently are likewise the most urbanized of provinces.) Hence, we used 15% as the lower bound and 40% as the upper bound for $\langle k \rangle$, the average proportion of cases due to travel.

The range of values between these upper and lower bounds for the three types of uncertainty were each divided into 21 equally-sized sections. Each range formed a side of a three-dimensional hybercube, and values were sampled from the hypercube according to the Latin hypercube algorithm, which is both computationally efficient and also yields a representative random sample of parameter space [59]. For each set of values thus sampled, simulations were run in order to obtain the best fit values for L, H, T and A and to determine the resulting fitted seroprevalence and predicted incidence.

Additional References for Additional File 3:

65. Richardson M, Elliman D, Maguire H et al: **Evidence base of incubation periods, periods of infectiousness and exclusion policies for the control of communicable disease in schools and preschools.** *Ped Inf Dis J* 2001, **20**:380-391.
